# Wine, Polyphenols, and the Matrix Effect: Is Alcohol Always the Same?

**DOI:** 10.3390/ijms25189796

**Published:** 2024-09-10

**Authors:** Elisabetta Miraldi, Giulia Baini, Marco Biagi, Giorgio Cappellucci, Alessandro Giordano, Federica Vaccaro, Alberto A. E. Bertelli

**Affiliations:** 1 Department of Physical Sciences, Earth and Environment, University of Siena, 53100 Siena, Italy; giulia.baini2@unisi.it (G.B.); giorgi.cappellucci@unisi.it (G.C.); alessandr.giordano@student.unisi.it (A.G.); f.vaccaro@student.unisi.it (F.V.); 2 Department of Food and Drug, University of Parma, 43121 Parma, Italy; marco.biagi@unipr.it; 3 Department of Biomedical Sciences for Health, University of Milan, 20133 Milan, Italy; alberto.bertelli@unimi.it

**Keywords:** wine, polyphenols, matrix, resveratrol, alcohol, non-alcoholic wine alternatives

## Abstract

While the number of publications on wine and health is steadily increasing, ranging from a molecular level to epidemiological studies, often with contradictory results, little attention has been given to a holistic approach to research, starting from the molecular level to arrive at pharmacological and medical conclusions. In this review, some unusual concepts are considered, such as the phytocomplex, the vehicle, and the Matrix effect. The concept of the phytocomplex is discussed, specifically the biological activities of Tyrosol, Hydroxytyrosol, and Resveratrol; indeed, the interactions among different molecules in herbal matrices provide a specific response. This is often markedly different from the response evoked by single constituents in the modulation of microbial populations in the gut, in intestinal stability and bioaccessibility, and, obviously, in inducing biological responses. Among the many alcoholic beverages which contain these molecules, wine has the most peculiar Matrix effect, which can heavily influence the bioavailability of the phytocomplex obtained by the fermentation processes that produce this beverage. Wine’s Matrix effect plays an instrumental role in improving the beneficial compounds’ bioavailability and/or in inhibiting alcohol metabolites’ carcinogenicity. Underestimation of the wine Matrix effect could lead to deceiving results, as in the case of dealcoholized wine or wine-compound-based nutritional supplements; alternatively, this can occur in the emphasis of a single component’s toxic activity, in this case, alcohol, ignoring the specific molecular-level protective action of other compounds (polyphenols) that are present in the same matrix. The dark side of the Matrix effect is also discussed. This review confirms the research recommendations made by the WHO Scientific Group, which suggests it is important “to investigate the possible protective effects of ingredients other than alcohol in alcoholic beverages”, considering that most recent studies seem not only relevant but also capable of directing future research towards innovative points of view that have so far been too neglected.

## 1. Introduction

The prevailing research methodology for plant-derived molecules still tends to isolate single active compounds, often overlooking their interactions with other molecules in an original matrix. This approach seeks to exploit specific and well-defined mechanisms at the sub-molecular pharmacophoric moiety level. Consequently, the choice of carriers, solvents, or excipients is frequently dictated by industrial or cost considerations. In contrast, research focused on herbal products—an official term for plant-based preparations used in human and animal health, defined as phytotherapy—aims to explore the complex interactions within a natural matrix. It evaluates the biological and pharmacological activities of compounds by considering them within their chemical context or, more commonly, by examining the pharmacodynamic (and pharmacokinetic) characteristics of the entire phytocomplex. This approach closely aligns with the methodological concept of holism, which recognizes the multiple chemical, technological, and pharmacological complexities involved.

Also, in food science, in particular in the topic of food and health, despite the fact that the role of the matrix should be rigorously prioritized, the classical lock–key molecular approach is still followed; one interesting example, focused on in this work, is wine and its characteristic polyphenols.

Wine is obtained through the fermentation of *Vitis vinifera* L. grapes and contains several hundred compounds, including polyphenols, in a water and ethanol (EtOH) matrix. Polyphenols are considered to hold primary responsibility for the health effects of fruit and vegetable consumption [1].

Dietary polyphenols are bioactive compounds that can especially be found in fruits and vegetables; they play a crucial role both for the plants that produce them and for the animals that consume them, as they are among the most important antioxidants that are capable of detoxifying UV-induced oxidative stresses and which are biologically active in mammal cells.

These compounds, which include flavonoids, phenolic acids, tannins, and lignans, are widely distributed in plants and contribute to the prevention of intense oxidative-stress-related dysregulations, such as cardiovascular diseases, type 2 diabetes, and certain types of cancer [2]. For instance, flavonoids, abundantly found in apples, onions, and tea, have been associated with a reduced risk of cardiovascular diseases through their ability to improve endothelial function and reduce the oxidation of LDL [3]. Additionally, the regular intake of polyphenol-rich fruits and vegetables has been correlated with improved gut health by modulating the gut microbiota and promoting the growth of beneficial bacteria [4].

Beyond their role in disease prevention, polyphenols have also been shown to exert protective effects against neurodegenerative diseases, such as Alzheimer’s and Parkinson’s, through their inhibition of neuroinflammation and oxidative stress in the brain [5]. Moreover, recent studies have highlighted the potential of polyphenols to improve metabolic health by enhancing insulin sensitivity and reducing inflammation in adipose tissue, thus playing a vital role in obesity management [6].

**Figure 1:**
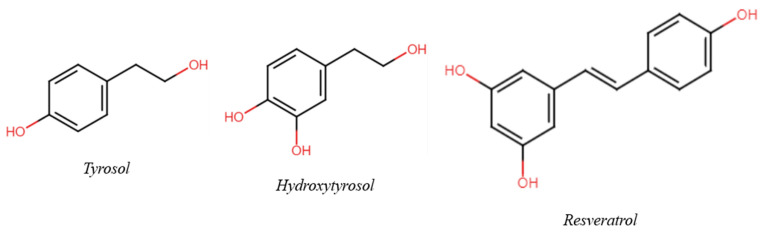
Chemical structure of the three main phenols/polyphenols of interest.

The aim of this review is to suggest a possible and useful variation in wine and health research to a holistic approach, evolving from classical molecular models to unusual concepts such as the Matrix effect and the phytocomplex. Taking three (poly)phenols present in wine as an example—Tyrosol (Tyr), Hydroxytyrosol (HTyr), and Resveratrol (Res) (Figure 1)—the consequences of respecting, disregarding, or ignoring their role in the context of the matrix are discussed; this is important not only for their effect on (poly)phenol research but also for the influence of such findings on decisions taken at a governmental level and, consequently, on public health. In selecting bibliographic references, priority was given to clinical and epidemiological studies. A short comment is also added. Reviews were selected from among the papers published in the last five years.

## 2. Fundamental Concepts for the Study of Plant-Derived Bioactive Compounds

### 2.1. The Importance of the Phytocomplex

The concept of the phytocomplex is the fundamental aspect of the use of plants for health, both in the food area and in pharmacology [7]. In fact, the interaction among different molecules in herbal matrices provides a specific response, clearly different from single constituents in the modulation of microbial populations in the gut [8], in intestinal stability and bioaccessibility [9], and, obviously, in evoking biological responses.

The modern, evidence-based phytotherapy is the conventional sector of pharmacotherapy in which herbal preparations are used for human (and animal) health. It is officially recognized by the World Health Organization (WHO) [10] but also, in Europe, by the European Medicines Agency (EMA) [11]. This field specifically takes into account the peculiar pharmacokinetics and pharmacodynamics of phytocomplexes in several medical contexts. Examples that explain the role of phytocomplexes and phytotherapy in a clear way can be found in *Ginkgo biloba* L. leaf standardized dry extract, obtained through water (H_2_O)/acetone extraction, which has a well-established use in the treatment of mild vascular dementia, only because of the multitarget activity of ginkgoflavones and diterpenes [12] or in *Hypericum perforatum* L. aerial parts’ standardized extracts, obtained through H_2_O/EtOH or methanol (MeOH) extraction, authorized in the market of almost all European and various countries outside Europe for the treatment of mild depressive symptoms because of the synergistic effect of flavonols, phloroglucinols, and hypericins in targeting Na^+^-channels and producing a non-specific inhibition of neurotransmitter re-uptake [13]. These two known examples consider two aspects worthy of special attention: (I) no single molecule of *G. biloba* and *H. perforatum*, even if at very high dosages, could be alternative to the phytocomplex; (II) the specific preparation is fundamental, and what is reported for an extract could be not applied to another one. This is also clearly indicated by the official monographs of medicinal plants issued by the WHO, EMA, and official pharmacopoeias.

As initially mentioned, the insight on medicinal plants and phytocomplexes is also directly related to food sciences, bearing in mind that safety, as well as the potential health benefits of food and food supplements, should be intrinsically related to the specific preparation used. This is so important that the European Commission requires the authorization of novel foods when simple or elaborate processing different from conventional methods is used [14]. In the context of plants, the direct consequence is that nutritional facts and health benefits along with therapeutic potential should not be preferred to phytocomplexes and preparations different from those tested and used.

This concept is worth exploring further through insights from studies on the wine matrix for research about wine and health.

The chemical composition of wine is peculiar. Starting from a raw water solution of sugars and hydrophilic and primary and secondary metabolites, most of them as glycosides, the fermentation process mainly operated by *Saccharomyces* spp. produces EtOH from sugars, stabilizes the acid environment, and consequently changes the proportion of soluble and insoluble metabolites. Furthermore, during the winemaking process, proanthocyanidins, gallotannins, and phenolic glycosides undergo natural degradation. Additionally, as detailed in Section 3.2, an increase in the lipophilic small phenolic content occurs, triggering an enrichment in flavonols in aglycone form, monomeric flavan-3-ols, anthocyanins, stilbenes such as Rsv, gallic acid and other phenolic acids with particular reference to Tyr, and hydroxycinnamic derivatives.

### 2.2. Vehicle, Matrix and Food Matrix

A pharmaceutical vehicle is a carrier or an inert medium used as a solvent (or diluent) in which the medicinally active agent is formulated and/or administered (Dictionary of Pharmacy, 1986); it is the most abundant excipient, and, as such, can influence various aspects of the pharmacokinetics of the active ingredient dissolved/dispersed in it. In the specific case of wine, EtOH represents the vehicle within this beverage Matrix.

The notion of a “food Matrix” has generated significant interest recently due to its implications for nutrition, food processing, and general wellness. In the scientific literature, however, the term is used with a range of connotations and, often, is literally associated with food itself.

The Food Matrix, defined by the United States Department of Agriculture (USDA) as “The nutrient and non-nutrient components of food and their molecular relationships, i.e., chemical bonds, to each other” [15], comprises all the micro-, meso-, and macroscopic structures in which the food elements are organized. The course of a chemical reaction can be influenced by several food-related elements; these effects can be collectively referred to as “the food matrix effect”. The biological variance that exists in the kinetics of the same reactions taking place in different meals is caused by the Food Matrix effect itself [16].

Among the many alcoholic beverages, wine has the most peculiar matrix, which can heavily influence the bioavailability of the phytocomplex obtained by the fermentation processes that produce this beverage.

## 3. Polyphenols: General Properties and Lipophilic Phenols in Wine

Among the countless substances produced by plants, polyphenols represent one of the main classes. Comprising molecules made up of one or more hydroxyl groups linked to aromatic rings, this class, due to the great number of possible variations in their structure, is one large family of different subclasses of molecules. These subclasses include flavonoids, phenolic acids, lignans, stilbenes, etc., some of them in turn divided into vast subcategories (e.g., anthocyanins and catechins in the subclass of flavonoids) [17].

The role of polyphenols in the physiopathology of the plant is prominent. In addition to attracting pollinators and having structural functions, they fulfill many defensive purposes. The antiradical, antioxidant, and antimicrobial activities protect the plant against the over-production of reactive oxygen species (ROS) (induced by UV radiation, drought, exposure to heavy metals, temperature stresses, attacks by herbivores or nematodes, etc.) or possible microbial invasions. Different molecules can interrupt the development of parasitic insects’ eggs and/or delay their growth [18,19].

Together with monounsaturated fatty acids (MUFAs), polyunsaturated fatty acids (PUFAs), and fibers, polyphenols are one of the main bioactive classes responsible for the beneficial health effects of the Mediterranean Diet. Such benefits mainly include great improvements to the cardiovascular system (actions on the endothelium, on thrombus formation, on the levels of blood lipids, anti-inflammatory and antioxidant activities) and reduction in the risk of type 2 diabetes (insulin sensibilization effect), cognitive disorders, and cancers (anti-neoplastic activity) [20,21,22].

### 3.1. Bioavailability

A substantial number of studies and research on polyphenols and their properties has confirmed the link between their intake through diet and the positive effects on the overall health state of an individual. Nevertheless, it is fundamental to consider the different forms by which polyphenols are found in food and all the possible modifications which the ingested phenolic pool is subjected to throughout the metabolization process.

It is known that polyphenols are scarcely bioavailable, considerably metabolized by the gut microbiota and quickly absorbed. The concentration of the native molecules in the blood is generally lower than that of their metabolic derivatives due to the additional biotransformation processes of the phenolic hydroxyl groups, exposed mainly to phase II reactions (sulfation, glucuronidation, methylation) [23].

Before evaluating the biological effects of polyphenolic metabolites, a thorough and precise characterization must be conducted due to the documented instability of many polyphenol molecules in cell culture media and the co-existence of multiple polyphenol species (unmetabolized, metabolized, and breakdown products) in these conditions. Currently, determining whether the biological effect being observed is a result of the cellular uptake of unmetabolized polyphenol compounds or that of newly formed metabolites [particularly relevant during long exposure studies (>24 h)] remains a major challenge in evaluating the in vitro biological activity of polyphenols [24].

The bioavailability of polyphenols varies greatly among them and, for some substances, also among different dietary sources. As demonstrated by Manach et al. [25], isoflavones and gallic acid are the polyphenols that humans absorb most readily, followed by quercetin glucosides, catechins, and flavanones with varying kinetics. Anthocyanins, galloylated catechins, and proanthocyanidins are the least well-absorbed types of polyphenols.

### 3.2. Polyphenols as Wine Matrix Constituents

Wine is a hydroalcoholic solution (the EtOH content rarely exceeding 14% *v*/*v*), which contains various substances, such as glycerol, polysaccharides, aldehydes and ketones, organic acids and esters, sugars, soluble proteins, vitamins, minerals, and polyphenols. The whole production procedure can deeply influence the specific composition of this beverage, from the type of grapes used to the specifics of the vinification process and storage. Certainly, contact with all the parts of the grape during maceration ensures a higher phenolic extraction in red wines than in white and rosé ones [26,27].

Wine, one of the foundations of the Mediterranean Diet [28], has been linked with the health benefits of this type of diet for a long time, in particular connected to its phenolic composition. The polyphenols which can be found in wine include many subcategories of flavonoids (flavones, flavonols, flavan-3-ols, flavanones, anthocyanins, etc.) together with various non-flavonoids, such as stilbenes, hydroxycinnamic, and hydroxybenzoic acids. Primary examples of molecules include catechin, epicatechin, and proanthocyanidins (flavan-3-ols), Rsv (stilbenes), anthocyanins, Tyr and HTyr, and phenolic acids (caffeic, coumaric, and ferulic acids and esters in the hydroxycinnamic group, gallic, vanillic and salicylic acids in the hydroxybenzoic one) [29,30].

### 3.3. Tyrosol, Hydroxytyrosol and Resveratrol

**Figure 2:**
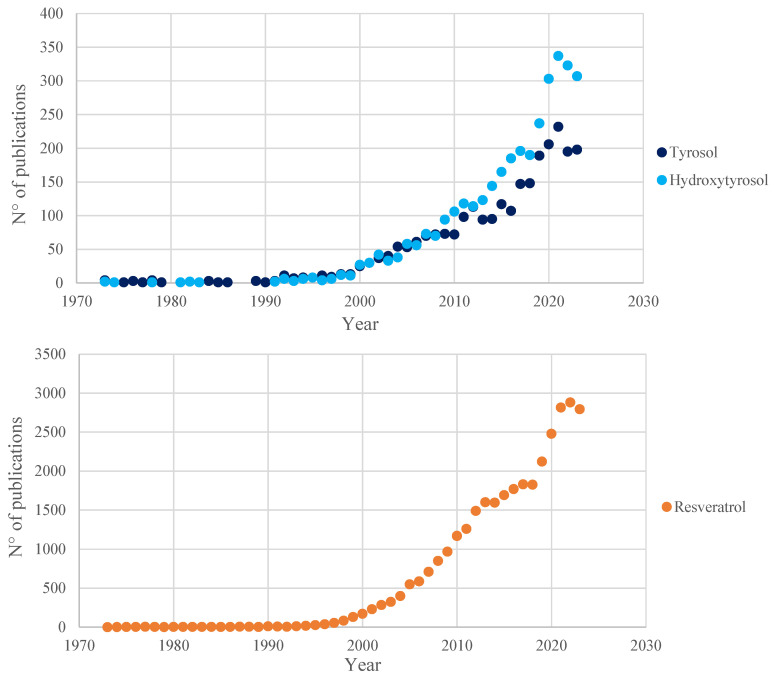
Number of publications, registered in the Scopus database (July 2024), in the past 50 years (1973–2023) related to the three main polyphenols analyzed in this review. Results obtained from the queries “tyrosol”, “hydroxytyrosol” and “resveratrol”, respectively.

In the vast phenolic pool found inside wine, Tyr, HTyr, and Rsv are of particular interest in the research field (Figure 2). Due to their peculiar lipophilicity, these three polyphenols are readily soluble in hydroalcoholic solutions, unlike most other molecules of the same class; this difference in the type of vehicle is one of the reasons for their higher bioavailability.

Their bioavailability is a central theme in the studies regarding their possible use as food supplements, mainly in the prevention of different pathologies. Polyphenols should be considered as an integral part of the wine Matrix: underestimating their role in this context could, thus, have important consequences.

#### 3.3.1. When Matrix Effect Is Respected: The Case of Tyrosol and Hydroxytyrosol

Even though Tyr and HTyr are extensively studied due to their significant levels in olive oil, their presence in wine could corroborate the positive health effects of Rsv and other phenolic compounds. These molecules have numerous health benefits and disease prevention properties [31,32]: studies conducted in vitro, in vivo, and in clinical trials have demonstrated their noteworthy bioactivity, both as isolated components and in a phytocomplex [33,34].

The simple phenol Tyr is found in fermented drinks like wine and beer, produced as a byproduct of tyramine metabolism [35]. Upon consumption, the human body can endogenously convert Tyr to HTyr [36]. Since in vitro research has shown that HTyr has a greater antioxidant activity than Tyr, this biotransformation is biologically important, taking into consideration also that the levels of HTyr in fermented alcoholic beverages are significantly lower than that of Tyr [37,38].

Research on the metabolites of these two phenolic alcohols is exiguous. The levels of absorption after oral administration of Tyr and HTyr are high, even quantitative in some cases [39], but influenced by the food matrices which convey the substances [40]. During digestion, the compounds undergo phase I and II reactions, while, if non-digested, they can also be metabolized by the gut microbiota. Htyr can be both metabolized and conjugated, while Tyr is mainly conjugated by phase II reactions. Some metabolites have been isolated and studied, some of them showing different biological actions, but a great part of them is yet unknown or left unstudied [41].

The correct evaluation of the HTyr role in the matrix, in the case of olive oil, led to the statement issued by the European Food Safety Agency (EFSA) that “the novel food, hydroxytyrosol, is safe under the proposed uses and use levels” [42]. Furthermore, even more important was the authorization for labelling HTyr on food products by the European Commission [43].

The Mediterranean Diet has been inscribed by UNESCO in the Representative List of the Intangible Cultural Heritage of Humanity since 2010 [44]. An interesting review by Santos-Buelga et al. [45] shows an updated version of the Mediterranean Diet Pyramid. This list of foods could be considered as an indication for a super-matrix, where both quality and quantity are suggested. Three matrices (wine, EVO, and olives) are mentioned for their contribution to phenols. Nevertheless, from the exhaustive list of natural ingredients in Table 1 from the same reference, it is clear that both HTyr and Tyr may vary in quantity. This causes the Matrix effect to vary based on the quality of the oils, with EVO as the best one. Furthermore, processing conditions such as de-bittering may interfere with the oil Matrix by lowering oleuropein levels, leading to lower Htyr levels.

In another review [46], an interesting map indicates that table olives and wine are the main contributors to the dietary HTyr intake in European countries. However, in the same publication, it is suggested that HTyr could be added to yogurt, sausages, cookies, smoothies, and even blood orange juice, raising uncertainty about how a liposoluble compound could work in those matrices, particularly the latter one.

#### 3.3.2. When Matrix Effect Is Disregarded: Resveratrol

Rsv is the most investigated molecule in the class of stilbenes. The identification of the molecule and the discovery of the connection of this compound with the beneficial effects of wine consumption on the cardiovascular system are well known [47], as well as its plausible biological role in the so-called “French paradox” [48].

After being taken orally as a pure compound, enterocytes absorb Rsv in enormous amounts; nevertheless, only a small portion (less than 1%) of this dietary ingredient enters the bloodstream and bodily tissues, mainly because of its efficient metabolism in the liver and the intestine [49]. Some of the most significant restrictions and difficulties associated with the in vivo use of Rsv include rapid absorption, low bioavailability, and low solubility in H_2_O [50].

Rsv is chemically stable at low temperatures, in both acidic [51] and anoxic [52] environments and in the dark [53], all conditions found in bottled wine.

To improve absorption efficacy, solubility, and relative bioavailability, various possibilities in the administration methodologies could be explored, such as, for example, the link with serum albumin [54] or the encapsulation in protein nanoparticles [55]. Likewise, the administration strategies should contribute to the “reservoir” effect to allow Rsv accumulation in tissues and its subsequent mobilization [56,57].

In one of the first kinetic studies in rats in which natural resveratrol in the red wine matrix was used, plasma resveratrol concentrations from 100 nM to 1 μM were detected [58], levels that are sufficient to explicate the cardioprotective activity [57]. In humans, after red wine intake, Rsv was easily absorbed and detected even in low-density lipoproteins (LDLs) [59]. It is, therefore, surprising to note the trend of increasing synthetic resveratrol dosage, not only in animals but also in humans [60,61]. The matrix effect in red wine containing resveratrol was already described as early as 1995 [62], just three years after the discovery of the French paradox [48]. The decision to disregard the Matrix effect in many research projects could make it rather difficult to overcome bioavailability problems.

An important contribution to the research on Rsv is given by the review of Visioli et al. [30]. Here, it has been suggested that, rather than trying to separate and study individual molecules, which would be a pharmacological approach, a valid alternative could be to test the effects of the whole polyphenol fraction. Possibly, the next step could be extending this concept by testing the same molecules in different matrices.

Rsv toxicity has been extensively reviewed by Shaito et al. [63] at the molecular, cellular, and tissue levels. As already mentioned, the main issue with Rsv is dosage-related toxicity. Except for plants from Chinese (*Veratrum formosanum* O. Loes., *Reynoutria japonica* Houtt.) or pre-Colombian (*Cassia quinquangulata* Rich.) traditional medicine, where Rsv in piceid form could reach 3% of dry material, the amount of natural Rsv in edible plants is commonly low, rarely reaching 1.5 mg/Kg. In addition, in *Vitis vinifera* L. fruits, the Rsv content is not higher, whereas in some wines, it could reach 5.8 mg/L [64].

Rsv consumption from wine or other medicinal herbs matrices began at least 2.500 years ago. Provided that only small dosages of Rsv are ingested from natural matrices, important toxic side effects should not occur.

This is consistent with the concept of hormesis. As explained in another recent review [65] on the molecular mechanisms of the health effects of plant polyphenols, hormesis is described as a process characterized by a biphasic response to the exposure to increasing the amount of a substance. This phenomenon is well known by researchers who seek to obtain a traditional dose/effect curve with certain polyphenols such as Rsv, obtaining a biphasic curve instead. As correctly pointed out by the authors, plant polyphenols are synthesized by plants to defend themselves against bacteria, fungi, and insects. To this end, even small amounts of polyphenols are sufficient and, consequently, small amounts are present in plants or plant-derived matrices, such as in wine.

## 4. Alcohol and Alcoholic Beverages

As suggested by the adjective, the multitude of existing alcoholic beverages is linked precisely by the presence of alcohol, in different concentrations (from 5 up to 40% *v*/*v* and even more), inside them. Due to its relatively high concentrations compared to other components and the heavy influence that this substance has on the organism, it is obvious that the physiological effects of EtOH play a key role among the consequences of the consumption of these drinks.

### 4.1. Absorption and Metabolism of Ethanol

Passive diffusion allows alcohol to enter the bloodstream from the digestive system. The duodenum and jejunum absorb most of the EtOH that is taken orally, so the rate at which the stomach empties is a significant factor in determining how quickly alcohol is absorbed. The amount of alcohol consumed, the presence or absence of food in the stomach (food delays gastric emptying and hence reduces EtOH absorption), factors that affect gastric emptying (meals high in fat, carbs, or protein are equally effective in delaying gastric emptying), and the rate of EtOH oxidation all affect the blood alcohol concentration. The rate at which the same amount of EtOH is absorbed when given in different alcoholic beverages does not vary significantly. Because alcohol is oxidized more readily than other nutrients, it is crucial to remember that calories from EtOH are obtained preferably instead of those from the metabolism of regular nutrients [66,67,68,69,70,71].

Less than 3% of the absorbed EtOH is expelled by sweat, urine, and breath, but more than 90% of it circulates throughout the body and is eventually delivered to the liver via the portal vein. Both oxidative and nonoxidative metabolic processes are used in the liver to break down alcohol. In two stages, the liver converts EtOH into acetate, which is used in the citric acid cycle. When drinking in moderation, specific alcohol-oxidizing enzymes usually drive the elimination of alcohol; when drinking excessively, non-specific enzymes may increase alcohol elimination even more. ADH is the primary and most specific alcohol-oxidizing enzyme, transforming EtOH into acetaldehyde. The enzyme ALDH efficiently transforms extremely poisonous acetaldehyde into innocuous acetate. In peripheral tissues, acetate is broken down into CO_2_, fatty acids, and H_2_O. The cytochrome (CY) P2E1 that is part of the microsomal ethanol-oxidizing system (MEOS) is expressed and activated more when alcohol consumption is excessive. Activated CYP2E1 stimulates the synthesis of acetaldehyde by producing ROS. Nonoxidative metabolism produces ethyl glucuronide (EtG), ethyl sulfate (EtS), PEth, and FAEEs from a minor portion of EtOH (<0.1%). These EtOH metabolites have a substantially longer half-life than EtOH itself [67,72,73,74,75,76,77,78].

### 4.2. Physiological Effects and Toxicity of Alcohol

Alcohol enters the brain and momentarily alters signal transduction. After moderate alcohol consumption, the neurotransmitters glutamate, dopamine, serotonin, and γ-aminobutyric acid (GABA) undergo the most significant short-term alterations. EtOH reduces glutamate activity and increases GABA activity, which promotes tranquility, relaxation, pleasure, and a reduction in tension. EtOH also causes a spike in serotonin and increases the release of dopamine, further enhancing the pleasant effects [79].

Alcohol metabolic byproducts harm the liver and are a major factor in the progression of alcohol-related liver diseases (ALDs), from alcoholic steatosis to alcoholic cirrhosis. Acetaldehyde is the most well-known harmful substance that results from the metabolism of EtOH. Direct interactions between acetaldehyde and DNA result in chromosomal damage and point mutations. Additionally, it forms acetaldehyde adducts via binding to a series of proteins, which alters the structure and function of the liver. These protein adducts increase oxidative stress and upregulate CYP2E1 expression. Furthermore, it has been shown by Holstege et al. [80] that protein adducts are important in the pathophysiology of different phases of ALD by promoting lipid accumulation, inflammation, and fibrosis. EtOH toxicity is also known to be caused by metabolites produced from nonoxidative pathways, such as phosphatidylethanol (PEth) and fatty acid ethyl esters (FAEEs), although the exact processes are still unknown [81,82,83,84,85,86].

#### The Dark Side of the Matrix Effect: Alcohol and Smoke

The toxic effects of ethanol consumption on the organism are also synergic with those of other harmful substances.

There is a strong dose–response association between alcohol intake and the development of head and neck cancer (HNC), making it an independent risk factor [87,88]. Acetaldehyde and alcoholic beverages are categorized as class I carcinogens [89]. After being metabolized, alcohol may contribute to HNC carcinogenesis directly or indirectly. The latter may occur, for instance, due to alcohol acting as a solvent for other potential carcinogens, such as those found in tobacco [90,91]. The risk between drinking beer, wine, or liquor and developing HNC is comparable, suggesting that ethanol, rather than other components in alcoholic beverages, is likely the most significant factor in determining HNC risk [92]. However, the risk of HNC was generally inversely correlated with wine consumption [93]. Numerous studies have already proven the strong correlations between smoking cigarettes and an elevated risk of HNC in general and all subtypes [94,95]. Furthermore, there seems to be a correlation between the risk of HNC overall and cigarette smoking status, frequency, and duration, and quitting smoking also lowers the risk of HNCs [96,97,98]. A multiplicative interaction between the categories of cigarette smoking and alcohol intake was established in the HNC as a whole [99,100]. Since alcohol can function as a solvent for carcinogens in cigarette smoke and increase the mucosa’s permeability to these carcinogens, the interaction effect between alcohol consumption and cigarette smoking is biologically plausible. As a result, the carcinogenic properties of both factors are likely to be enhanced in the presence of one another [101,102].

### 4.3. When the Matrix Effect Is Ignored: Alcohol Beverages Specificity

In the search for the biological motivation for the pro-carcinogenic effect of alcoholic beverages, acetaldehyde resulting from the metabolism of EtOH has been indicated as a cause of malignant tumors, particularly those affecting the esophagus [103]. However, the specificity of the distinct types of alcoholic beverages is not considered since, in most studies, usually no distinctions are made. Therefore, there is a tendency to also consider wine as responsible for the onset of esophageal tumors through a mechanism of action mediated by acetaldehyde. Nevertheless, several studies on the upper digestive tract not only do not confirm this hypothesis but even suggest a protective effect of wine in these pathologies [104,105,106,107,108,109]. A recent review about moderate red wine intake also pointed to its association with a positive effect on mortality and dementia, as well as specific malignancies, like non-Hodgkin lymphoma, and circulatory diseases. Meanwhile, the correlation for other medical conditions was insignificant [110].

Is there a biological plausibility for this protective effect of wine? It would seem so, since the polyphenols in the wine itself are responsible. As already known, acetaldehyde can react in wine with other phenolic-type components to the detriment of the quality of the product [111]. At least thirteen polyphenols present in wine have been shown to be “potent inhibitors of the mammary tissue microsomal pathway of EtOH metabolism to acetaldehyde” [112]. Three other polyphenols (epicatechin, epicatechin gallate, and epigallocatechin) extracted from the fruit of *Diospyros kaki* L.f., the persimmon, “attenuate acetaldehyde-induced DNA double-strand breaks by scavenging acetaldehyde” [113]. The three mentioned polyphenols are also present in wine [114]. Furthermore, epigallocatechin can silence the activation of hepatic stellate cells induced by acetaldehyde, which plays a key role in hepatic fibrogenesis [115].

The possible correlation between wine consumption and oral cancer is also the subject of a recent review [116]. While wine, as other alcoholic beverages, may contain a certain amount of acetaldehyde, the oral microflora appears to be the main origin of acetaldehyde levels in saliva. Wine appears to act as an oral cavity cancer preventer, with the chemo-preventive activity in a great number of wine components being reviewed with biochemical, in vitro, in vivo, and in human studies. Studies on the chemopreventive activity of the wine Matrix should be considered in the future.

Rsv decreases acetaldehyde generation and increases the metabolism of acetaldehyde to acetic acid by enhancing acetaldehyde dehydrogenase 2 (ALDH2) in cultures of human peripheral lymphocytes [117]. It must be underlined that the activation of ALDH2 is also of cardiovascular interest as it “reduces ischemic damage to the heart” and may be beneficial for patients subjected to cardiac ischemia [118]. Rsv and other flavonoids showed promising results in pre-clinical trials by lowering blood pressure, increasing vascular health, and attenuating left ventricular remodeling and hypertrophy, while enhancing its functionality. These polyphenols can also have a beneficial impact on several cardiovascular risk factors, including vascular inflammation, diabetes, and lipid profiles, while also exhibiting strong anti-atherogenic properties [119].

The prevention of cardiovascular disease has been linked to procyanidins (tetra-epicatechin-gallate, procyanidin trimer-, tetramer- and pentamer-gallate). These polyphenols are particularly abundant in red wines from south-west France (Gers) and Sardinia (Nuoro) and can be linked to the peculiar increased longevity in these two regions [120]. Furthermore, other studies showed the possible preventive action of red wine against weight gain and health hazards associated with obesity [121,122], clearly also connected with those affecting the circulatory system, as opposed to spirits and beer [123,124].

Recent epidemiological research confirms the cardiovascular protective effect of moderate wine consumption [125], although for some researchers, it seems to be limited to the prevention of ischemic heart disease [126]. Moreover, in the latter cited study, the authors underline the importance of distinguishing between the several types of alcoholic drinks, a distinction that is rarely considered in most of this type of research. In a recent systematic analysis for the Global Burden of Disease Study 2020, the authors themselves admit that a limitation in the report is precisely that of not having differentiation between the various types of alcoholic beverages [127].

The recommendations for research by the WHO Scientific Group, which suggests “to investigate the possible protective effects of ingredients other than alcohol in alcoholic beverages” [128], considering the most recent studies, seem not only relevant but also capable of directing future research towards innovative points of view that have so far been overly neglected.

## 5. In Vivo Studies on Polyphenol Biological Activities

The modest bioavailability and high biotransformation of most polyphenols in the organism cast doubt on the possibility that their antioxidant activity can play a major role in their in vivo effects. However, in vitro activity is well substantiated and has been repeatedly linked in the literature to its health effects [129].

Even though in vivo polyphenols have a mild but significant direct antioxidant activity [130], other factors are now thought to be involved in their biological effects. They might, for instance, regulate the expression of genes and intracellular signaling pathways that are essential to the protection and functioning of cells [131,132]. Additionally, there is mounting evidence that the interactions between gut microbiota and polyphenols play a vital role in explaining the health advantages of polyphenol ingestion. In fact, the range of substances that can have a biological impact was substantially expanded by the discovery of metabolites produced by the microbiota [133].

A comprehensive revision including in vivo investigations on the impact of proanthocyanidins on the lipid metabolism was conducted by Bladé et al. [134]. Numerous dietary anthocyanins, including cyanidin-3-rutinoside and pelargonidin-3-glucoside, as well as the flavanones hesperetin and naringenin and their corresponding in vivo metabolites, have been shown to be able to penetrate the blood–brain barrier (BBB) [135,136]. Regretfully, the results of these numerous investigations into neuroprotective processes typically use flavonoids in their natural state rather than those that are more likely to penetrate the bloodstream [137].

According to in vitro and in vivo studies, phenolics from olive oil can activate the 5′ adenosine monophosphate-activated protein kinase (AMPK), which then inhibits the mTOR signaling pathway [131]. This pathway is involved in the control of several adipose tissue activities, such as lipid metabolism, thermogenesis, and adipogenesis. Moreover, it alters cell cycle progression, hypoxia signaling, autophagy, mitochondrial biogenesis, and functioning [138]. Due, in part, to their high polyphenol content, virgin-olive-oil-rich Mediterranean diets were found to be beneficial in lowering a number of inflammatory markers in intervention studies, including tumor necrosis factor (TNF) α, interleukin (IL) 6, chemokines like the monocyte chemoattractant protein (MCP), etc. [45,139,140].

There is a multitude of research on in vivo human studies about the connections between wine, phenolic components, and health (Table 1). A study by Welch et al. [141] looked at how various wine flavonoids affected the bone mineral density (BMD) of more than three thousand female twins from the United Kingdom. Anthocyanins and flavones had good effects on hip and spine BMD, and total flavonoid intake had a positive association with spine BMD, implying that phenolics may be advantageous for human bone health [142].

**Table 1:** References from different meta-analyses, referring to human studies, which highlight the positive effects of wine/grape product consumption.

Reference	N° of Studies	Treatment	Results	Outcomes
[143]	12	Wine (242–94.308 mg/L/die)	↓ CICAM-1 ↓ VCAM-1 ↓ TNF-α ↓ CCR2 ↑αMβ2 (Mac-1)	Improvement of the markers associated with atherosclerotic inflammation in healthy patients, but not in those with CVD
[144]	4	Red wine vs. white wine and/or other alcoholic beverages	/	Red wine decreased the risk of Alzheimer’s Disease
[145]	4	Grape extract (700–1400 mg/die)	↓ blood pressure ↑ FMD	Improvement of vascular health, particularly in at risk human populations
[146]	11	Wine (0–84.9 g/die)	/	There is not a statical significant correlation between wine consumption and prostate cancer
[147]	9	Wine (118–300 mL/die)	↓ total cholesterol ↓ diastolic blood pressure	Improvement of some cardiovascular parameters in type 2 diabetes mellitus patients
[148]	99	Red wine/grape extract (100–2000 mg/die) Red wine (250–400 mL/die)	↓ total cholesterol ↓ blood pressure ↑ HDL-C ↑ FMD ↓TAGs	Improvement of cardiovascular health Support in the treatment of metabolic disorders
[149]	6	Red wine (moderate consumption, max one glass/die)	/	Decrease in the risk of developing Non-Hodgkin Lymphoma, epithelial ovarian cancer, prostate cancer, lung cancer, breast cancer, esophageal adenocarcinoma (Barret’s esophagus)

↑—Increase; ↓—Decrease.

Although wine polyphenols should not be viewed as direct antioxidants in vivo, under certain circumstances, they may act in that way. Lipid peroxides are formed in the stomach during digestion, particularly after consuming red meat, and can reach concentrations of up to mM [150,151]. Foods and drinks high in polyphenols, such as extra virgin olive oil and red wine, can scavenge lipid peroxides and stop and lessen the development of such peroxides.

By blocking the sphingosine kinase 1/sphingosine phosphate pathway, green tea and wine polyphenols limit prostate cancer cell proliferation, both in vitro and in vivo, according to a study that employed a grapevine extract [152].

It has been demonstrated that resveratrol increases bone growth and inhibits bone loss. By using a number of molecular mechanisms that have all been demonstrated in in vitro studies, it prevented osteoclast differentiation and bone loss [153]. Its preventive benefits in bones are confirmed by in vivo animal investigations [154,155].

Scientists studying human health have recently become interested in trans-epsilon-viniferin, also known as ε-viniferin, a trans-resveratrol dimer. Red wine is its primary food source, with the highest concentrations found roughly around1 mg/L [156]. Numerous studies have examined the biological effects of viniferin over the past fifteen years, and its anti-inflammatory and antioxidant qualities have been documented [157]. Certain reports claim that the biological properties of ε-viniferin are often even higher than those of resveratrol. It has been shown to have positive benefits in both in vivo and in vitro models of cancer, obesity, disorders connected to obesity, and neurological or cardiovascular diseases [158].

## 6. When Natural Vehicle in Wine Is Targeted: Non-Alcoholic Alternatives to Wine

If, in the future, the consumption of wine is also penalized from a regulatory point of view due to the alcohol content, various scenarios will have to be hypothesized in which wine is replaced by other, non-alcoholic products.

One feasible alternative could be grape juice, which, however, is ineffective as a matrix in the absorption of two polyphenols of interest like quercetin and Rsv, both in animals and humans. It was observed that when administration occurs by adding 10% EtOH or whiskey, polyphenols are present both in plasma and urine, pointing to a good level of absorption of the molecules [159].

A perhaps more rational substitute would be dealcoholized wine, but the experimentation of this product in post-menopausal women gave underwhelming results [160], while unmodified red wine instead reduces the risk of cardiovascular diseases [161]. A study in humans on different circulatory functions, with particular attention to flow-mediated dilatation (FMD), reaches the same conclusions, confirming once again the functioning of red wine but not of the dealcoholized one [162].

Another product used as a wine substitute is red wine extract (RWE), whose formulation is highly variable in quantity and quality. Comparing the action of RWEs in humans, the results are conflicting and with completely different FMD values [163,164].

Considering wine as a phytocomplex, there must be an interaction between its components, in particular polyphenols. This has been observed both in vitro [165] and in vivo [166]. However, even if a perfect correspondence with the wine’s phytocomplex was possible in the formulation of the extracts, the role of EtOH always remains indispensable, according to some authors, as a synergistic element [167] or even considered as the sole factor responsible for the protective effect of wine [168].

The synergistic effect between the components of RWEs was observed on an intracellular level and is considered “not the result of a single phytochemical activity, but that of all the components of wine extracts that crossed cell membranes” [169]. It should be noted that, in this study, the extracts are dissolved in a hydroalcoholic Matrix before use to facilitate penetration. The presence of EtOH is also a principal factor in the bioavailability of Tyr in humans.

Many doubts, therefore, remain, not only on the effectiveness but also on the logic with which RWEs are formulated: for example, the effect mediated by the Sirtuin 1 enzyme (expressed from the gene SIRT1) [170] should have been better calibrated by giving less importance to Rsv but also considering only malvidin [171], already present in RWEs.

A recent review [64] analyzes, in separate sections, new European Labeling rules of wine and viticultural and enological aspects of a red wine from Catalunya, Spain. At first glance, the two subjects seem completely different, but reading carefully the text reveals a connection. Even though the selected red wine production area is a small one, wines from five different micro-areas have been produced and analyzed, showing significative differences among them. Of particular importance are the levels of procyanidins for their acetaldehyde scavenging activity. From the labeling rules section, it seems that polyphenols, as well as other wine components, are not mentioned at all. If the details on the wine Matrix are not disclosed, at least partially, the question arises as to how it is possible that a consumer can be adequately informed.

## 7. Conclusions and Future Perspectives

The concept of the Matrix effect in food is not a widespread one. Usually the pharmacological aspect prevails, leading to the isolation of specific molecules to be sold as drugs or food supplementation. This procedure, being already quite difficult with a normal phytocomplex, becomes even more puzzling when products of natural fermentation are present, like EtOH and bacterial/yeast byproducts in the wine Matrix. On the other hand, the wine Matrix plays an instrumental role in improving beneficial compound bioavailability or in inhibiting alcohol metabolite carcinogenicity. Indeed, wine specificity and scientific research have already been recommended, even by intergovernmental organizations [172].

This review suggests a new path for a holistic study of wine, a product of plant origin with a rich phytocomplex, by starting from molecular bases of some wine components to reach biological/medical conclusions. Unfortunately, this conceptual approach is present only in a limited number of articles, if compared to the thousands of publications on wine and health.

So far, the “French paradox” has been discussed in an enormous medical/epidemiological bibliography in which the resveratrol protective role has been emphasized as a single molecule. In the present review, the authors followed the opposite way of thinking, and failures in targeting a single molecule or in artificially modulating the vehicle have been discussed. In the vast phenolic pool found inside the wine, Tyr, HTyr, and Rsv stand out due to their lipophilicity; indeed, they are readily soluble in hydroalcoholic solutions, in contrast to most of the other molecules of the same class. This difference in the type of vehicle is one of the reasons for their higher bioavailability, which is a central theme in studies with the aim of researching their possible use as food supplements in the prevention of different pathologies. Polyphenols should be considered as an integral part of the wine Matrix. Underestimating their role in this context could have important consequences.

The interaction between various molecules in herbal matrices provides a specific response, often markedly different from the one induced by single constituents, in the modulation of microbial populations in the gut, in intestinal stability and bioaccessibility, and, obviously, in evoking biological responses. Among the many alcoholic beverages which contain these molecules, wine has the most peculiar matrix, which can heavily influence the bioavailability of the phytocomplex obtained by the fermentation processes that produce this beverage. The wine Matrix plays an instrumental role in improving beneficial compound bioavailability or in inhibiting alcohol metabolite carcinogenicity. Therefore, it is very important to know the consequences of respecting, disregarding, or ignoring the Matrix effect. Underestimation of the wine Matrix effect could lead to deceiving results, as in the case of dealcoholized wine or wine-compound-based nutritional supplements, or in emphasizing a single component’s toxic activity, in this case, alcohol, ignoring the specific protective action at the molecular level of other compounds (polyphenols) present in the same matrix. It is suggested that the specificity of wine should always be taken into consideration by public health authorities.

Future perspectives should consist of recommendations from the competent authorities to suggest investigating the possible protective effects of ingredients other than alcohol in alcoholic beverages, taking into consideration that the most recent studies seem not only relevant but also capable of directing future research towards innovative points of view that have so far been too neglected.

With the public health interest as the objective, wine specificity should be finally given the consideration it deserves.

## Abbreviations

ADH	Alcohol dehydrogenase
ALD	Alcohol-related liver disease
ALDH	Acetaldehyde dehydrogenase
AMPK	5′ adenosine monophosphate-activated protein kinase
BBB	Blood–brain barrier
BMD	Bone Mineral Density
CO_2_	Carbon dioxide
CYP2E1	Cytochrome P450 isoform 2E1
EFSA	European Food Safety Agency
EMA	European Medicines Agency
EtG	Ethyl glucuronide
EtOH	Ethanol
EtS	Ethyl sulfate
FAEE	Fatty acid ethyl ester
FMD	Flow-Mediated Dilatation
GABA	Gamma-aminobutyric acid
H_2_O	Water
HDL-C	High-Density Lipoprotein-Cholesterol
HNC	Head and Neck Cancer
HTyr	Hydroxytyrosol
IL	Interleukin
LDL	Low-Density Lipoprotein
MCP	Monocyte Chemoattractant Protein
MeOH	Methanol
MEOS	Microsomal ethanol-oxidizing system
MUFAs	Mono-Unsaturated Fatty Acids
PEth	Phosphatidylethanol
PUFAs	Poly-Unsaturated Fatty Acids
ROS	Reactive Oxygen Species
Rsv	Resveratrol
RWE	Red Wine Extract
TAG	Triacylglycerol
TNF	Tumor Necrosis Factor
Tyr	Tyrosol
USDA	United States Department of Agriculture
UV	Ultraviolet
WHO	World Health Organization
